# Thermal management of prismatic lithium-ion battery using mist cooling with single and multiple injections: a numerical study

**DOI:** 10.1038/s41598-026-54239-z

**Published:** 2026-07-17

**Authors:** Pranshu Gadepalli, Rhik Banerjee, Mohan Sushmitha, Kottayat Nidhul

**Affiliations:** Manipal Institute of Technology, Manipal Academy of Higher Education, Manipal, Karnataka India

**Keywords:** Battery thermal management system, Mist-assisted air cooling, Prismatic lithium-ion battery, High C-rate discharge, Two-phase CFD simulation, Energy science and technology, Engineering, Materials science

## Abstract

Thermal management of high-discharge prismatic lithium-ion battery packs remains challenging due to their large planar heat-transfer surfaces, strong streamwise thermal gradients, and stringent temperature uniformity requirements. This study numerically investigates mist-assisted air cooling as an enhancement to conventional forced air cooling for a high-discharge (5 C) prismatic LiFePO₄ battery pack. A transient three-dimensional CFD framework that incorporates detailed spray dynamics, droplet breakup, evaporation, and two-way phase coupling is employed to compare pure air cooling with single- and multiple-mist injection configurations. Results indicate that pure air-cooling leads to a peak cell temperature of approximately 62 °C, accompanied by significant thermal nonuniformity. Single mist injection reduces the maximum temperature by 2–3 °C but exhibits limited downstream effectiveness due to vapour accumulation. In contrast, multiple mist injection achieves a peak temperature reduction of nearly 10 °C with reduced non-uniformity in temperature. Vapour mass-fraction contours reveal that distributed injection sustains evaporation along the entire flow path, mitigates boundary-layer redevelopment, and ensures uniform latent heat absorption. Transient analysis further demonstrates improved thermal stability under sustained discharge. The findings establish multiple mist injection as a lightweight, energy-efficient, and effective cooling strategy for high-rate prismatic battery thermal management.

## Introduction

Growing concerns over resource depletion, sustainability, and environmental pollution have accelerated the shift toward electric vehicles (EVs), which offer higher energy efficiency, lower emissions, and reduced maintenance compared to traditional internal combustion engine vehicles^1^. Central to EV performance is a reliable and high-energy-density storage system, for which lithium-ion batteries have emerged as the leading technology. Moreover, when powered by renewable energy sources, EVs have the potential to lower greenhouse gas emissions by nearly 40%^2^. The battery pack is the prime mover for EVs in terms of its operation and performance. Today, EVs employ lithium-ion (Li-ion) batteries owing to their high energy and power density, and fast charging capability with longer cycle life^3^. Even with sustainable prospects, lithium-ion battery-powered EVs have not been widely used owing to limited life and capacity loss, resulting in higher cost and even safety issues with thermal runaway^4^.

As a Lithium-ion battery involves the conversion of chemical energy into electric energy, heat is generated. An appropriate amount of heat is favourable for the rate of electrochemical reactions. However, excessive heat generation and accumulation in the battery may lead to non-homogeneous temperature distribution and an increase in temperature. Whereas the maximum temperature of Lithium-ion batteries should be limited to 60 °C under adverse operating conditions^5^. It was observed that the capacity of battery-operated devices above 55 °C decreased to 30% within 100 charging-discharging cycles^6^, with a state-of-health (SOH) lower than the standard value of 80%^7^. At 60 °C, the battery impedance increased after 140 cycles, accompanied by a decrease in power^8^. Beyond 80 °C, large amounts of heat were generated due to the exothermic reaction between the electrodes and electrolyte, resulting in more severe thermal deterioration known as thermal runaway, which elevated the battery temperature^9^. Once the thermal runaway is triggered, due to a substantial temperature rise (> 100 °C), the metal oxide within the positive electrode decomposes and releases oxygen, increasing the likelihood of an explosion in the battery^10,11^. In addition, localised overheating can also deteriorate the performance if a large temperature gradient is built up in cells. In a practical scenario, the temperature non-homogeneity of the battery pack should preferably be less than 5 °C^12,13^. Hence, battery thermal management systems (BTMS) are a critical component to remove the heat generated from cells to ensure optimal performance and lifespan^14–16^.

In the initial stages, researchers employed air cooling as it was a prominent solution in the domain of electronics component cooling. With different cell arrangements and fan locations, it was observed that the best cooling is achieved with a cubic cell configuration and a fan at the top and bottom of the duct. Furthermore, a hexagonal arrangement of cells was proposed for optimal space utilisation with optimised inter-cell distance^17^. The effect of a heat pipe coupled with a low air flow rate was reported as an efficient method to limit the battery temperature within the optimal range. Since the air flow rate requirement was lower, the parasitic power consumption and noise levels from the cooling fans were minimised^5^. A two-directional airflow cooling system was suggested, as previous studies reported that cells at the centre of the battery pack were at a higher temperature compared to cells at the front.

For a constant flow rate, the effects of longitudinal and transverse spacing of cells were investigated for both aligned and staggered cell configurations. It was reported that higher transverse spacing leads to higher battery temperatures for both configurations^18^. Forced air cooling for a prismatic cell has been reported to show that the air flow channel layout has a substantial impact on the temperature characteristics of the battery pack^19^. To improve temperature homogeneity, a secondary inlet was provided by adding a plenum for an axial air flow cooling system, which changes the direction of flow and eliminates recirculation eddies. Compared to the conventional method, a 4% and 39% reduction in maximum temperature and non-homogeneity in temperature distribution were obtained^20^. The impact of thermal interface material on varying thicknesses with air cooling was investigated. A 25% reduction in the maximum temperature of the battery pack was achieved, with the material thickness having a negligible impact^21^.

Using two-dimensional numerical simulations, it was reported that reciprocating flow improves temperature homogeneity by 4 K for a reciprocating duration of 120 s^22^. For 100s of reciprocating flow, temperature homogeneity was improved by 3 K, along with a 38% reduction in coolant flow, using a control strategy with reduced cooling time compared to unidirectional flow^23^. Using adjacent channels with a diathermanous material, the cooling performance of air flow in the opposite direction was reported to have a lower maximum temperature, along with improved temperature homogeneity, owing to counterflow heat exchange through the partition^24^. With varying reciprocating timings, an optimal value of 50% of the depth of discharge (DOD) was obtained, owing to better temperature homogeneity and a lower maximum temperature compared to 30% and 80%, for a 3 °C discharge rate^25^. The reduction in temperature is attributed to disturbances of the boundary layer on cells and subsequent heat redistribution. Due to its simple and lightweight structure, air cooling is commonly used in electric vehicles (EVs), offering the advantages of being economical and environmentally friendly. Recent numerical studies have shown that air-cooling performance in lithium-ion battery packs can be enhanced by introducing vortex generators, which induce secondary flows, leading to improved heat transfer and reduced peak cell temperatures. However, these improvements come at the cost of increased pressure drop and parasitic power, underscoring the inherent limitations of air cooling for high heat-flux battery applications^26^. Further, due to lower heat capacity and thermal conductivity, air cooling fails to achieve higher cooling efficiency and maintain temperature homogeneity at higher discharge rates^27,28^.

Cooling of next-generation EV battery packs with ultrafast charging will be challenging with air cooling, and liquid cooling is the solution, of which direct cooling or immersion cooling is preferred^29,30^. Recent experimental investigations have demonstrated the significant thermal and safety advantages of liquid-immersion cooling for large-format lithium-ion batteries. Static and dynamic immersion cooling can significantly suppress temperature rise and improve thermal uniformity of high-capacity prismatic battery modules across a wide ambient temperature range, including extreme cold and hot conditions^31^. Complementary studies further reported that immersion cooling effectively prevents thermal runaway propagation in large energy-storage modules under severe abuse conditions, highlighting its role in enhancing thermal safety^32^. In addition, accurate characterisation of battery heat-generation behaviour under varying temperature-rise regimes has been reported, providing essential inputs for evaluating advanced thermal management strategies^33^.

While improving temperature homogeneity, immersion cooling reduces thermal contact resistance, thereby requiring a simpler cooling design than indirect liquid cooling. A compact BTMS was designed for lithium-ion pouch cells by varying the immersion depth from 3 cm to 13.2 cm (corresponding to the total cell height) at a discharge rate of 2 C, using transformer oil as the coolant. With improvement in temperature homogeneity, the maximum temperature of the battery pack decreased by 32.4% compared to air cooling^34^. At a 5 C discharge rate, a temperature homogeneity of 6.6 °C was achieved, with a maximum temperature of 35 °C, using a combination of immersion and forced convection^35^.

Numerical investigations comparing different dielectric fluids for the immersion cooling of lithium-ion battery packs have shown that the coolant’s thermo-physical properties strongly influence temperature homogeneity and peak cell temperature. The study reported that selecting an appropriate dielectric fluid can significantly reduce the maximum temperature rise and intercell temperature gradients, demonstrating that immersion cooling is an effective but fluid-dependent thermal management strategy^36^. Recent numerical investigations into dielectric immersion cooling of prismatic lithium-ion battery modules have demonstrated that effective thermal control can be achieved even at very small inter-cell spacings (< 1 mm), maintaining maximum cell temperatures within 25–40 °C and ensuring temperature non-uniformity of less than 5 °C. The study further demonstrated that the associated pumping power is negligible relative to the battery energy (< 0.01%), highlighting immersion cooling as a thermally robust yet system-intensive solution for densely packed prismatic cells^37^. An experimental comparison of cylindrical and prismatic cells under ester-oil immersion cooling reveals a substantial temperature reduction compared to air cooling, although prismatic cells exhibit higher tab-level non-uniformity due to geometry-induced flow stagnation^38^.

This numerical investigation compares multiple dielectric fluids for immersion-cooled battery packs and shows that the thermo-physical properties of the coolant strongly influence peak temperature and temperature homogeneity, emphasising that immersion cooling performance is highly fluid-dependent [Effect of various dielectric fluids on temperature homogeneity of Li-ion battery packs in an energy-efficient novel immersion cooling design^36^. The authors also explored the performance of nanofluid for cooling high-discharge cells and reported a maximum reduction of 7 K with a temperature homogeneity of 5 K^39^. A large-format prismatic LiFePO₄ battery pack (280 Ah) study demonstrates that immersion cooling effectiveness depends strongly on structural parameters and flow distribution, highlighting scalability challenges and design sensitivity for EV-scale prismatic battery systems^40^. This experimental study evaluated immersion-cooled lithium-ion battery modules under varying ambient temperatures, demonstrating that immersion cooling maintains acceptable thermal control across different climates, although cooling effectiveness degrades at elevated ambient conditions^31^.

Among the various cooling techniques employed in BTMS, forced-air cooling is a preferred method among battery manufacturers due to its simple structure, lightweight design, and lower initial cost. However, for cells with higher discharge rates, air cooling, due to its lower heat capacity, cannot effectively maintain cells within the optimal temperature range while ensuring uniform distribution. Hence, recent research has focused on addressing these shortcomings, with the cooling technique that uses the coolant’s latent heat of evaporation gaining attention. Evaporative cooling, in which a two-phase mixture of fine droplets is introduced into the mainstream air, offers better thermal management owing to the superior thermal properties of liquid compared to those of air. For 3 C, BTMS using mist cooling (fine droplets of water sprayed into the air) with a 5 g/s flow rate and a 3% mist fraction was able to maintain battery temperatures below 40 °C, with a variation of less than 5 °C. The mist loading fraction plays a substantial role in determining cooling performance. Furthermore, the results indicate that mist cooling enhances thermal performance by 45% with lower power consumption compared to air cooling systems^45^. For 1.8 C, a polyethene fibre coating was used in conjunction with evaporative cooling to achieve a lower maximum temperature of 32 °C, thereby maintaining the simplicity of the battery pack and reducing energy consumption^41,43^. Using water spray as pre-cooling, various parameters such as water flow rate, droplet size, air velocity and ambient temperature were studied, and the result indicated that for an ambient temperature of 35 °C, air velocity above 2 m/s and water flow rate of 0.0002 kg/s was sufficient to keep the battery pack within the optimal range of operational temperature^46^. Three modes of cooling, namely natural convection, forced convection, and direct evaporative cooling (DEC), were studied, and the comparison indicated that DEC reduces the inlet air temperature, thereby enhancing heat dissipation from the cells. As the temperature was above 5 °C, reciprocating air cooling was employed, resulting in an improvement. Further, it was also suggested that DEC can be a promising technique in high ambient air temperatures due to increased cooling potential^45^. With a combination of heat pipe and air cooling, the condenser part of the heat pipes cooled using a water mist reported a drop in maximum temperature of the battery by 29 °C, compared to natural convective cooling for an ambient temperature of 40 °C and 1.9 °C^47^. Jute fibre as a sustainable, cost-efficient cooling medium in BTMS was explored using evaporative cooling and forced convection. With the integration of moistened jute in the airflow path, the cooling potential increased, resulting in lower power consumption, especially in hot ambient conditions^48^. Using a dielectric liquid (hydrofluoroether) spray with forced air cooling, a reduction of 6 °C in maximum temperature was achieved, along with a temperature homogeneity of 4 °C. The quantity of spray and arrangement of the spray nozzle had a substantial impact on the temperature distribution of the battery^43^. A jet impingement cooling BTMS with a target plate was proposed for hybrid electric vehicles, utilising mist cooling principles. The study was conducted at varying air temperatures (20 °C to 31 °C) and relative humidities (60% to 96%). It was observed that mist spray cooled the target plate by 0.8 K for a mist flow rate of 5.5 mg/s. Further, for varying mist flow rate (15 to 40 mg/s), the ambient air temperature can be reduced by 7 K^49^. Hybrid cooling with the use of hydrophilic fibres was explored for BTMS using various fibre materials, of which tissue paper fibre proved more effective due to its rapid wetting and water-holding capacity relative to others. A 42% improvement in temperature uniformity was achieved compared to conventional forced air cooling^50^.

As the automotive industry prefers forced air cooling for thermal management due to its compact design and lower cost compared to direct or indirect liquid cooling, which can pose challenges such as leakage in direct liquid cooling or a bulky design in indirect cooling. Hence, acknowledging the lower cooling capacity of air and the increased pumping power requirement at higher flow rates, research is directed towards using air cooling at medium flow rates in conjunction with liquid phase change, owing to its ability to dissipate more heat. In this regard, liquid droplets are introduced into the main air stream as fine droplets or mist, allowing them to absorb heat from the air stream as they evaporate. As the specific heat capacity of water is higher than that of air, a higher cooling capacity will be retained from the inlet to the exit of the cooling domain, increasing the cooling capacity with a reduced streamwise temperature gradient, a potential aspect in battery cooling, without a significant trade-off in cost and space constraints. However, the literature indicates that research on battery cooling via mist evaporation is in its early stages, with a potential gap in exploring this approach across varying climatic conditions, particularly in practical application scenarios.

Despite extensive research on air- and liquid-based battery thermal management systems, effective cooling of high-discharge prismatic lithium-ion battery packs remains challenging due to large planar heat-transfer surfaces, strong streamwise thermal gradients, and stringent uniformity requirements. While mist-assisted air cooling has shown promise in enhancing heat removal through latent heat absorption, existing studies are largely limited to single-point injection strategies, providing limited insight into spatial evaporation control within densely packed prismatic modules. In particular, the influence of injection strategy on vapour distribution, boundary-layer redevelopment, and inter-cell thermal uniformity under severe operating conditions has not been systematically examined. Motivated by these gaps, the present study numerically investigates mist-assisted air cooling of a high-discharge (5 C) prismatic LiFePO₄ battery pack, with a specific focus on comparing single- and multiple-mist injection configurations. A transient three-dimensional CFD framework incorporating detailed spray dynamics, droplet breakup, evaporation, and two-way phase coupling is employed to elucidate the coupled thermo-fluid mechanisms governing cooling performance. The novelty of this work lies in demonstrating how distributed mist injection sustains evaporation along the entire flow path, mitigates downstream thermal degradation, and significantly improves temperature uniformity without increasing the primary airflow rate. This provides a physically grounded justification for mist-assisted cooling as a lightweight and energy-efficient enhancement to conventional air cooling for prismatic battery packs. The manuscript is organised as follows: Sect. 2 describes the battery model, governing equations, numerical methodology, and validation. Section 3 presents and discusses the thermal performance of air cooling, single mist injection, and multiple mist injection configurations. Finally, Sect. 4 summarises the key findings and outlines conclusions and future research directions.

## Methodology

### Battery pack and cell description

**Fig. 1:**
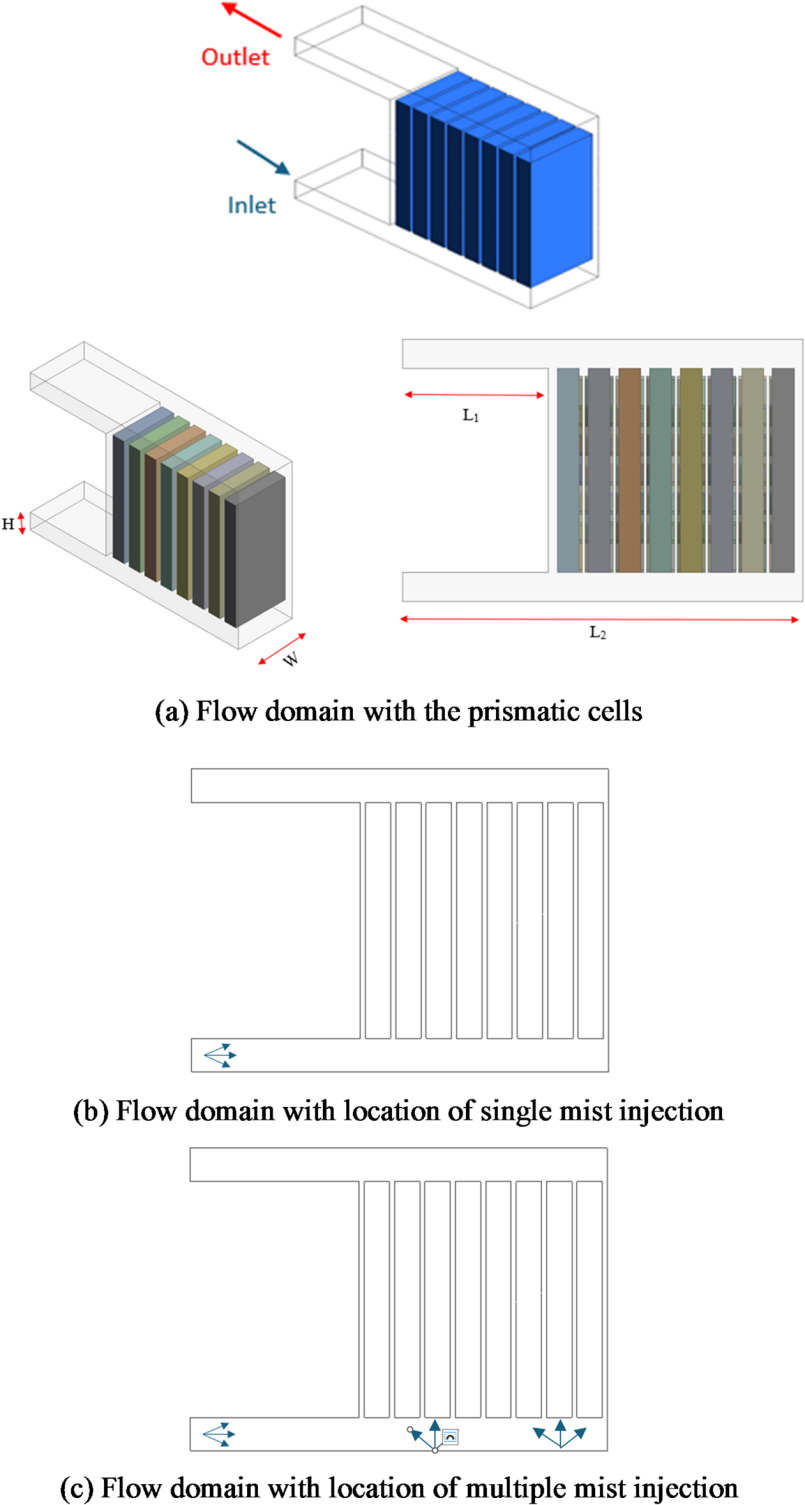
Schematics of the flow domain with cells of the battery pack and mist injection locations.

**Fig. 2:**
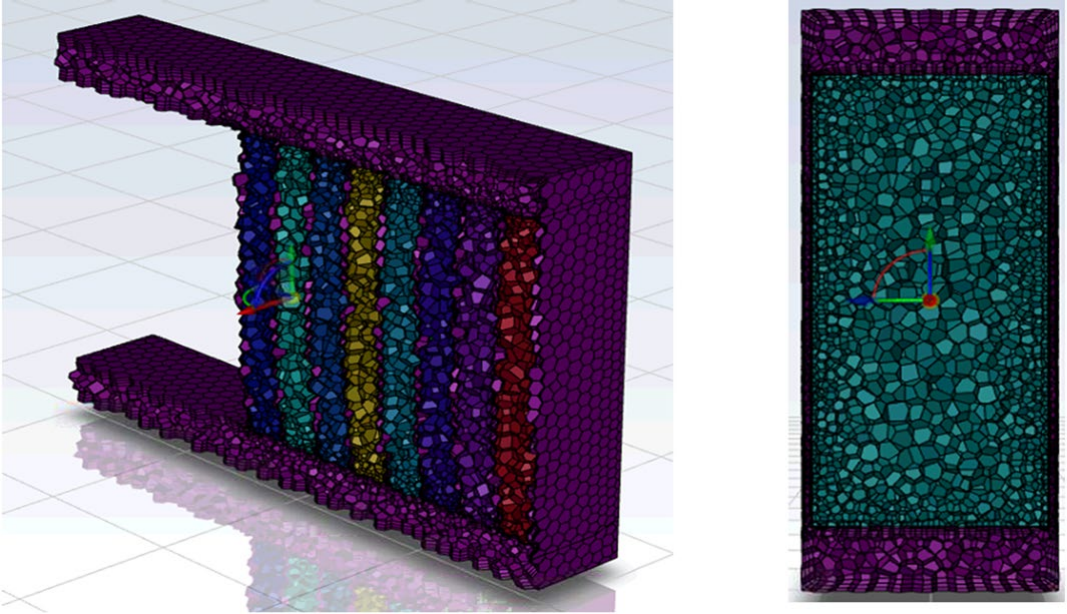
Domain discretised using polyhedral cells with dense grids near the walls.

**Fig. 3:**
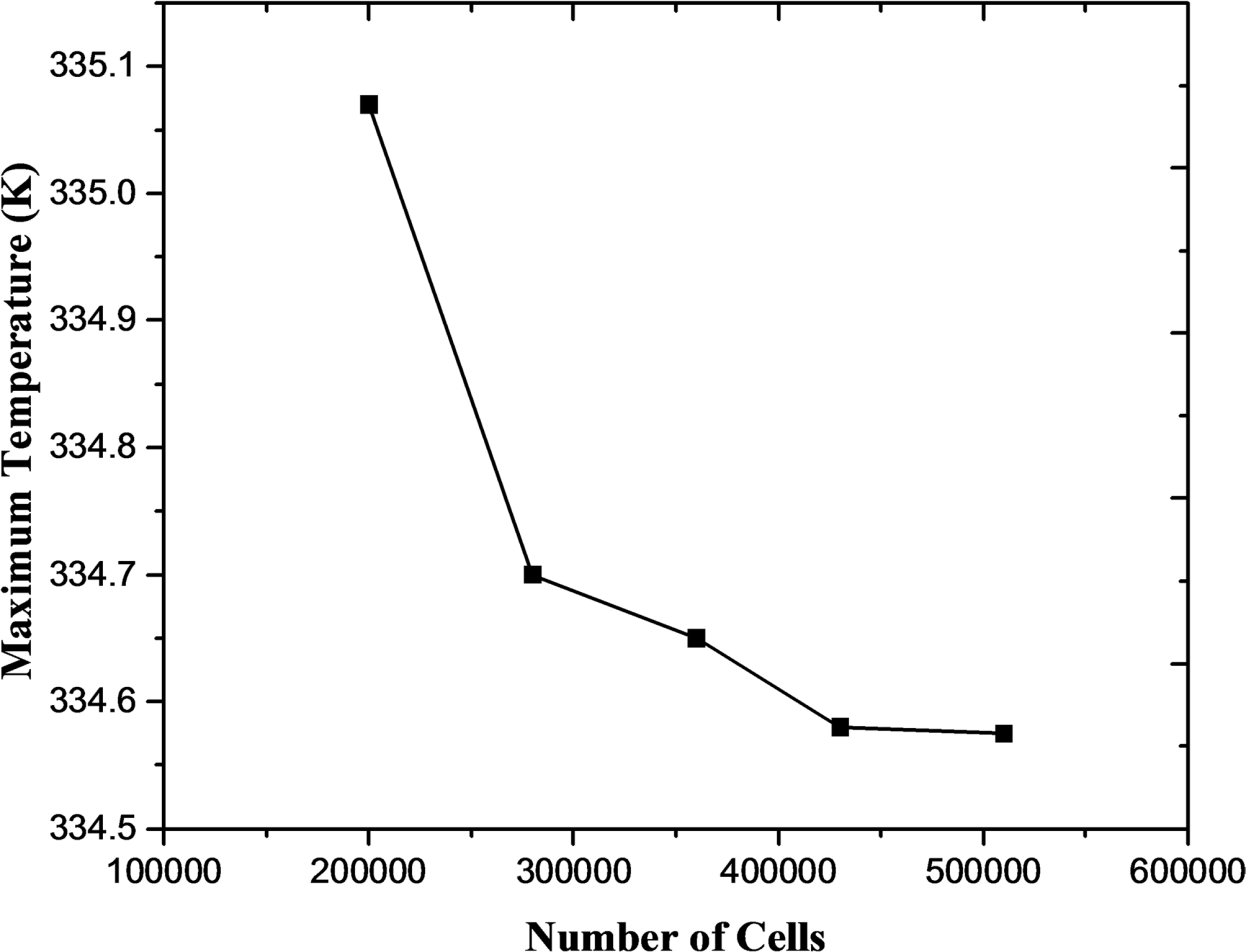
Grid independence study.

An 8S1P battery pack of prismatic (LiFePO4) lithium-ion cells is modelled to assess cell-level heat generation and subsequent thermal response (Fig. 1). Each cell has a capacity of 10 Ah and a nominal voltage of 3.2 V, consistent with prior high-rate discharge studies involving similar cylindrical geometries and the thermophysical properties are displayed in Table 1^51^ With a domain as shown in Fig. 1, the effect of mist cooling is studied for multiple injections. The domain has a width (W), duct height (H), and hydraulic diameter of 76 mm, 20 mm, and 52.4 mm, respectively. Spacing between the cells of the battery pack is 3 mm. The dimensions of the domain are mentioned in Table 2. Polyhedral cells are used to discretise the fluid domain (Fig. 2) while ensuring that sufficient grid is employed near the walls to keep the yplus close to 1. To optimise computational resources and ensure that the results do not vary with the elements, a grid-independent study was conducted, and the variation in the maximum temperature of the battery was examined to determine the optimal grid size (Fig. 3). The optimal grid size was found to be nearly 420,000 cells.

**Table 1:** Thermophysical properties of the battery.

Parameter	Prismatic (LiFePO_4_) cell
Configuration	8s1p
Pack voltage (V)	25.6 V
Pack capacity (A.h)	10 Ah
Discharge rates (C)	5
Thermal Conductivity (W/m. K)	15.6 (k_x_), 1.4 (k_y_), 15.6 (k_z_)
Dimensions of cell (mm)	140 × 65 × 15

**Table 2:** Dimensions of the domain.

Dimension	Value (mm)
H	20
W	76
Hydraulic Diameter	52.4
L1	100
L2	274

With the experimental^52^ heat generation for a discharge of 5 C, a user-defined function (UDF) is employed to model the transient heat generation rate.

The transient heat-conduction equation used for obtaining the thermal field inside each cell is

1 $$\:\rho\:{C}_{p}\frac{\partial\:T}{\partial\:t}=\nabla\:.\left(k\nabla\:T\right)+\dot{q}\left(t\right)$$

Where T is the temperature (K), k is the anisotropic thermal conductivity tensor (W/mK), $\:\dot{q}\left(t\right)$ is the volumetric heat generation source (W/m^3^).

Due to the lack of experimentally measured voltage-DoD characteristics curves for the cell used in the present study, using volumetric heat generation rate is consistent with the established modelling practice in battery thermal simulation literature^44,53–55^. Further, this method was used due to several reasons:

1. At higher C-rates, the irreversible heat, that is, I^2^R term dominates the heat generation.
2. The bulk cell temperature to internal heat source spatial variation sensitivity is low.
3. The MSMD (Multi-Scale, Multi-Dimensional) method requires much higher computational demand relative to volumetric heat generation simulations.

In addition, benchmark studies have shown that the output of MSMD and the volumetric heat models differ by only 5.8%^56^ at 1 C and 0.5% at 1.5 C, indicating diminishing deviations with increasing discharge rates. Further, numerous studies have reported that while MSMD improves spatial temperature resolution, peak temperature and thermal management metrics remain essentially unchanged^53,55,57^. Furthermore, a detailed spatial temperature distribution is necessary only for thermal runaway or hot spot prediction studies, rather than for evaluating cooling performance and comparing it with conventional systems, due to the higher computational requirements of two-phase simulations^42^.

### Problem description

Thermal management of the battery pack, as mentioned in Sect. 2.1, is studied using mist cooling at a discharge rate of 5 C, with air serving as the primary cooling medium with a flow rate of 0.108 kg/s. Water was introduced as a fine mist in a conical spray pattern at a loading fraction of 5% with both air and water streams maintained at an inlet temperature of 298 K. The influence of water droplet diameter is 0.1 mm. A single mist injection at the bottom of the duct is compared to the cooling performance with multiple mist injections. The thermal response of the system depends strongly on the fluid properties, including density, viscosity, specific heat capacity, and thermal conductivity. These properties for both air and coolant were obtained from the ANSYS Fluent material property database. Air is commonly used as a cooling medium because it is lightweight and easy to handle, owing to its low density and viscosity. However, its relatively low specific heat capacity limits its ability to remove heat efficiently, often requiring higher flow rates that may increase pressure losses. To enhance the overall cooling capability and overcome the inherent limitations of air, a fine water mist is introduced into the airflow.

### CFD analysis

A three-dimensional transient CFD framework in ANSYS Fluent is used to examine the flow behaviour and heat transfer characteristics of the cooling design. The governing equations are solved using the finite-volume method with an explicit formulation. Pressure–velocity coupling is achieved through the SIMPLE scheme, and spatial discretisation is performed with a second-order upwind method to ensure accurate resolution of gradients. Turbulent flow regions are modelled using the RNG k-ε turbulence model with enhanced wall treatment, while laminar zones are captured using the laminar viscosity model. Radiation heat transfer is not accounted in this study^58^. Convergence is controlled by setting the residual limits for the continuity and energy equations to 1 × 10 ^−^⁶. Key output variables, such as maximum cell temperature, are monitored throughout the transient solution. The conservation equations solved in this work are provided in Eqs. (2)–(4).

Continuity Equation:

2 $$\:\frac{\partial\:\rho\:}{\partial\:t}+\:\nabla\:.\left(\rho\:\overrightarrow{u}\right)=0$$

Momentum Equation:

3 $$\:\rho\:\frac{Du}{Dt}=-\nabla\:p+\mu\:{\nabla\:}^{2}\overrightarrow{u}+\:\overrightarrow{{S}_{m}}$$

Energy Equation:

4 $$\:\left(\rho\:{c}_{p}\right)(\frac{\partial\:T}{\partial\:t}+\left(\overrightarrow{\mathrm{u}\:.}\nabla\:)T\right)=\left(k\right){\nabla\:}^{2}T+\:\varphi\:$$

The spray dynamics are modelled using the Discrete Phase Model (DPM), in which the air flow is treated as a continuous Eulerian phase, and the water droplets are tracked individually using a Lagrangian approach. Droplets are injected with a constant diameter, specified velocity, and a defined cone-spray pattern. Their trajectories are influenced by drag, gravity, and momentum exchange with the surrounding turbulent air flow, with turbulence-particle interactions accounted for through stochastic tracking based on the local turbulence fields. Droplet breakup is modelled using the Taylor Analogy Breakup (TAB) model, which allows droplets to deform and fragment in response to aerodynamic forces. Evaporation of the droplets is treated using the Species Transport model, in which liquid–vapour mass transfer is computed from local thermodynamic conditions and vapour concentration gradients. The governing equations for particle motion, turbulence coupling, breakup, and species-based evaporation are presented below^59^.

#### Governing equations for particle motion

The trajectory of the particle is predicted by integrating the force balance of the particle in a Lagrangian reference frame. The force balance equation is given by:

5 $$\:{\boldsymbol{m}}_{\boldsymbol{p}}\frac{\boldsymbol{d}\overrightarrow{{\boldsymbol{u}}_{\boldsymbol{p}}}}{\boldsymbol{d}\boldsymbol{t}}=\:{\boldsymbol{m}}_{\boldsymbol{p}}\frac{\overrightarrow{\boldsymbol{u}}-{\overrightarrow{\boldsymbol{u}}}_{\boldsymbol{p}}}{{\boldsymbol{\tau\:}}_{\boldsymbol{r}}}+\:{\boldsymbol{m}}_{\boldsymbol{p}}\frac{\overrightarrow{\boldsymbol{g}}({\boldsymbol{\rho\:}}_{\boldsymbol{p}}-\boldsymbol{\rho\:})}{{\boldsymbol{\rho\:}}_{\boldsymbol{p}}}+\:\overrightarrow{\boldsymbol{F}}$$

Where,

$$\:{m}_{p}\:\mathrm{i}\mathrm{s}\:\mathrm{t}\mathrm{h}\mathrm{e}\:\mathrm{p}\mathrm{a}\mathrm{r}\mathrm{t}\mathrm{i}\mathrm{c}\mathrm{l}\mathrm{e}\:\mathrm{m}\mathrm{a}\mathrm{s}\mathrm{s},$$

$$\:\overrightarrow{u}\:\mathrm{i}\mathrm{s}\:\mathrm{t}\mathrm{h}\mathrm{e}\:\mathrm{c}\mathrm{o}\mathrm{n}\mathrm{t}\mathrm{i}\mathrm{n}\mathrm{u}\mathrm{u}\mathrm{m}\mathrm{m}\:\mathrm{p}\mathrm{h}\mathrm{a}\mathrm{s}\mathrm{e}\:\mathrm{v}\mathrm{e}\mathrm{l}\mathrm{o}\mathrm{c}\mathrm{i}\mathrm{t}\mathrm{y},$$

$$\:{\overrightarrow{u}}_{p}\:\mathrm{i}\mathrm{s}\:\mathrm{t}\mathrm{h}\mathrm{e}\:\mathrm{p}\mathrm{a}\mathrm{r}\mathrm{t}\mathrm{i}\mathrm{c}\mathrm{l}\mathrm{e}\:\mathrm{v}\mathrm{e}\mathrm{l}\mathrm{o}\mathrm{c}\mathrm{i}\mathrm{t}\mathrm{y},$$

$$\:\rho\:\:\mathrm{i}\mathrm{s}\:\mathrm{t}\mathrm{h}\mathrm{e}\:\mathrm{c}\mathrm{o}\mathrm{n}\mathrm{t}\mathrm{i}\mathrm{n}\mathrm{u}\mathrm{u}\mathrm{m}\mathrm{m}\:\mathrm{f}\mathrm{l}\mathrm{u}\mathrm{i}\mathrm{d}\:\mathrm{d}\mathrm{e}\mathrm{n}\mathrm{s}\mathrm{i}\mathrm{t}\mathrm{y}\:$$

$$\:{\rho\:}_{p}\:\mathrm{i}\mathrm{s}\:\mathrm{t}\mathrm{h}\mathrm{e}\:\mathrm{p}\mathrm{a}\mathrm{r}\mathrm{t}\mathrm{i}\mathrm{c}\mathrm{l}\mathrm{e}\:\mathrm{d}\mathrm{e}\mathrm{n}\mathrm{s}\mathrm{i}\mathrm{t}\mathrm{y},\:$$

$$\:{\tau\:}_{r\:}\:\mathrm{i}\mathrm{s}\:\mathrm{t}\mathrm{h}\mathrm{e}\:\mathrm{p}\mathrm{a}\mathrm{r}\mathrm{t}\mathrm{i}\mathrm{c}\mathrm{l}\mathrm{e}\:\mathrm{r}\mathrm{e}\mathrm{l}\mathrm{a}\mathrm{x}\mathrm{a}\mathrm{t}\mathrm{i}\mathrm{o}\mathrm{n}\:\mathrm{t}\mathrm{i}\mathrm{m}\mathrm{e},$$

and $\:\overrightarrow{F}$ which is an additional force term including the virtual mass force and the force due to a pressure gradient.

The momentum transfer between the discrete phase and continuous phase is computed by checking the change in the momentum of the particle as it passes through each control volume. This momentum change is computed as

6 $$\:F=\sum\:\left(\frac{18\mu\:{C}_{D}Re}{{\rho\:}_{p}{d}_{p}^{2}\hspace{0.17em}24}\hspace{0.17em}\left({u}_{p}-u\right)+{F}_{\mathrm{other}}\right)\hspace{0.17em}\dot{{m}_{p}}\hspace{0.17em}{\Delta\:}t$$

Where, µ = viscosity of the fluid, $\:{{\uprho\:}}_{p}$ = density of the particle, $\:{{\uprho\:}}_{p}$ = diameter of the particle, Re = relative Reynolds number, $\:{\mathrm{u}}_{p}$ = velocity of the particle, u = velocity of the fluid, $\:{\mathrm{C}}_{d}$ = Drag coefficient, $\:\dot{{m}_{p}}$ = mass flow rate of the particles, Δt = time step, $\:{\mathrm{F}}_{other}\:=$ Other interaction forces.

#### Particle-phase interaction models

Unsteady particle tracking with high-resolution tracking was employed to accurately track the particle trajectory. The particle time step size was set to match the fluid time step size, and the maximum number of steps was limited to 2000 to ensure that every particle is tracked until it leaves the domain or evaporates. The thermophoretic force is included in the additional force term. It is a force experienced by the particles suspended in a gas of high temperature gradient in a direction opposite to that of the gradient. It is given by the following equation:

7 $$\:\overrightarrow{F}=\:{D}_{T,p}\frac{1}{T}\nabla\:\:\mathrm{T}$$

Where, $\:{D}_{T,p\:}$ is the thermophoretic coefficient which was determined by the correlation given by Talbot. The relation is as follows, assuming the droplet is a sphere, and the fluid is an ideal gas.

8 $$\:{D}_{T,p}=\:\frac{6\pi\:{d}_{p}{\mu\:}^{2}{C}_{s}(K+{C}_{t}Kn)}{\rho\:(1+3{C}_{m}Kn)(1+2K+2{C}_{t}Kn)}$$

Where, K_n_ is the Knudsen number, K is the ratio of continuum phase thermal conductivity and particle thermal conductivity,

$$\:{C}_{s}=1.17$$

$$\:{C}_{t}=2.18$$

$$\:{C}_{m}=1.14$$

T is the local fluid temperature,

µ is the fluid viscosity.

The staffman’s Lift force acting on the particle due to shear is considered in the additional force term.

9 $$\:\overrightarrow{F}=\:{m}_{p}\frac{2K\sqrt{v}\rho\:{d}_{ij}}{{\rho\:}_{p}{d}_{p}{\left({d}_{lk}{d}_{kl}\right)}^{\frac{1}{4}}}\left(\overrightarrow{u}-\:{\overrightarrow{u}}_{p}\right)$$

Where K = 2.594 and $\:{d}_{ij}$ is the deformation tensor.

Two-way turbulence coupling was enabled to account for particle effects on the mainstream turbulent quantities. Droplet stochastic collision, coalescence and breakup models were also employed.

#### Water vapour transport equation

The transport equation for water vapor is given by:

10 $$\:\frac{\partial\:}{\partial\:{x}_{i}}\left(\rho\:{u}_{i}{m}_{v}\right)=\:\frac{\partial\:}{\partial\:{x}_{i}}\left(\rho\:{D}_{AB}\frac{\partial\:{m}_{v}}{\partial\:{x}_{i}}\right)+\:{S}_{m}$$

Where, $\:\rho\:$ is density, $\:u\:$is velocity, $\:{m}_{v}$ is the mass fraction of water vapor, And $\:{S}_{m}$ is the source term for mass.

11 $$S_{m} = \frac{{\Delta m_{p} }}{{m_{{p0}} }}\frac{{\dot{m}_{{p0}} }}{{dV}}$$

12 $$\:{F}_{i}=\:\sum\:\left(\frac{18\mu\:{C}_{D}{Re}_{D}}{24{\rho\:}_{p}{D}_{p}^{2}}\right)\left({u}_{p,i}-{u}_{p}\right){\dot{m}}_{p}{\Delta\:}T$$

13 $$\:{\dot{q}}^{m}=\:\left(\frac{{\stackrel{-}{m}}_{p}}{{m}_{p0}}{c}_{P,p}{\Delta\:}{T}_{p}+\:\frac{{\Delta\:}{m}_{p}}{{m}_{p0}}\left({-h}_{fg}+\:{\int\:}_{{T}_{ref}}^{{T}_{p}}{C}_{P,v}dT\right)\right)\frac{{\dot{m}}_{p0}}{dV}$$

$F_{i} \:and\:\dot{q}^{m}$ are the momentum and energy source terms added to the respective equation to model the interaction between the continuous and discrete phase.

#### Governing equations for droplets

The governing equation for the droplet mass momentum and energy (evaporation) is given as follows:

*Mass Equation*:

14 $$\:\frac{d{m}_{p}}{dt}=\:-{\:h}_{m}{(\rho\:}_{v.s}-{\rho\:}_{v}{)A}_{p}$$

Where, $\:{A}_{p}$ is the surface area of the droplet, The subscript ‘p’ represents the droplet and $\:{h}_{m}$ is the mass transfer coefficient given by:

15 $$\:Sh=\:\frac{{h}_{m}{D}_{p}}{{D}_{AB}}=2.0+0.6{Re}_{D}^{0.5}{Sc}^{1/3}$$

where Sh is Sherwood Number.

*Momentum Equation*

16 $$\:\frac{d{u}_{p}}{dt}=\:\frac{18\mu\:}{{\rho\:}_{p}{D}_{p}^{2}}\frac{{C}_{D}{Re}_{D}}{24}(u-\:{u}_{p})$$

$\:{D}_{p}$ is the droplet diameter,

$\:{C}_{D}$ is the drag coefficient, and.

$\:{Re}_{D\:}$is the relative Reynold’s number given by the following equation,

17 $$\:{Re}_{D}=\:\frac{\rho\:{D}_{p}|{u}_{p}-u|}{\mu\:}$$

Energy Equation

18 $$\:{m}_{p}{c}_{P,p}\frac{{dT}_{p}}{dt}=h{A}_{p}\left(T-{T}_{p}\right)+\:{h}_{fg}\frac{d{m}_{p}}{dt}$$

Where the heat transfer coefficient ‘h’ is defined by the following correlation:

19 $$\:Nu=\:\frac{h{D}_{p}}{k}=2.0+0.6{Re}_{D}^{0.5}{Pr}^{1/3}$$

The air was modelled as a continuous phase, with water droplets in the form of a mist serving as a secondary, discrete phase. These droplets are released in parcels and tracked using the Lagrangian approach. The interaction between the two phases was enabled, which computes the effects of these particles’ trajectories on the continuous-phase flow, as well as heat and mass transfer between them. Unsteady particle tracing was enabled with a time step of 0.001s, and the particles were injected at every particle time step. The maximum number of tracking steps was set to 2000 to ensure that all particles are fully tracked until they leave the domain or evaporate. Each particle was tracked and updated once per fluid flow time step (at the beginning of every time step).

The step length factor was set to 5, and accuracy control was enabled, which automatically adjusts the step length factor in every integration step to achieve the specified tolerance of 1e-4. High-resolution tracking was enabled by decomposing cells into smaller tetrahedra, enabling more accurate and robust tracking. Radiation effects were ignored in the heat transfer between the particles and the surroundings. The staffman lift force was enabled in the interaction between the particles and the continuous phase, which calculated the lift force acting on the particles due to the shear forces acting on them. Two–way turbulence coupling was enabled to account for particle effects on the continuous-phase flow structures. The stochastic collision, coalescence and breakup model was enabled, and the number of breakup parcels was set to 5.

DPM boundary conditions are as follows: The inlet and outlet of the domain were set to escape, the walls of the domain were set to trap, and a reflect boundary condition was applied to the cell walls. Particle wall heat transfer was enabled so that, in the event any particles come into contact with the hot cells, the heat transfer is computed while they remain in contact. Fluent assumes the particle deforms into a cylindrical shape when it comes into contact with a wall and computes heat transfer using the following relation.

20 $$\:\frac{d}{dt}\left({m}_{p}{c}_{p}{T}_{p}\right)=\frac{{k}_{l}{A}_{\mathrm{cont}}}{h}\left({T}_{w}-{T}_{p}\right)$$

Where,

$\:{\mathrm{m}}_{p}$ = droplet particle mass, $\:{\mathrm{c}}_{p}$ = droplet specific heat, $\:{\mathrm{T}}_{p}$ = particle temperature, $\:{\mathrm{A}}_{cont}$ = effective particle-wall contact area, h = particle centre-point to wall distance, $\:{\mathrm{k}}_{l}$ = droplet thermal conductivity, $\:{\mathrm{T}}_{w}$ = wall temperature.

### Validation

**Fig. 4:**
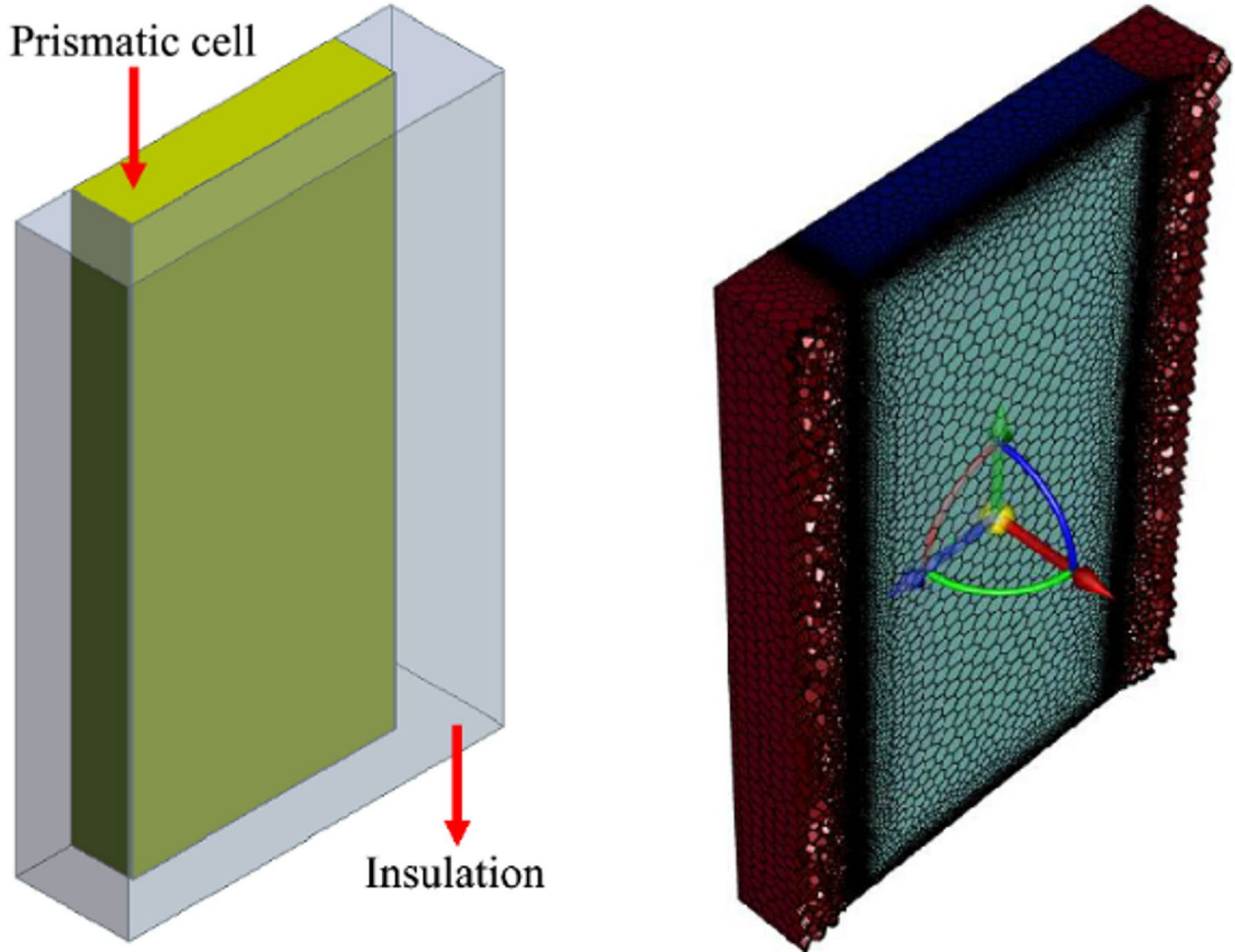
Validation domain and mesh displaying the polyhedral cells.

**Fig. 5:**
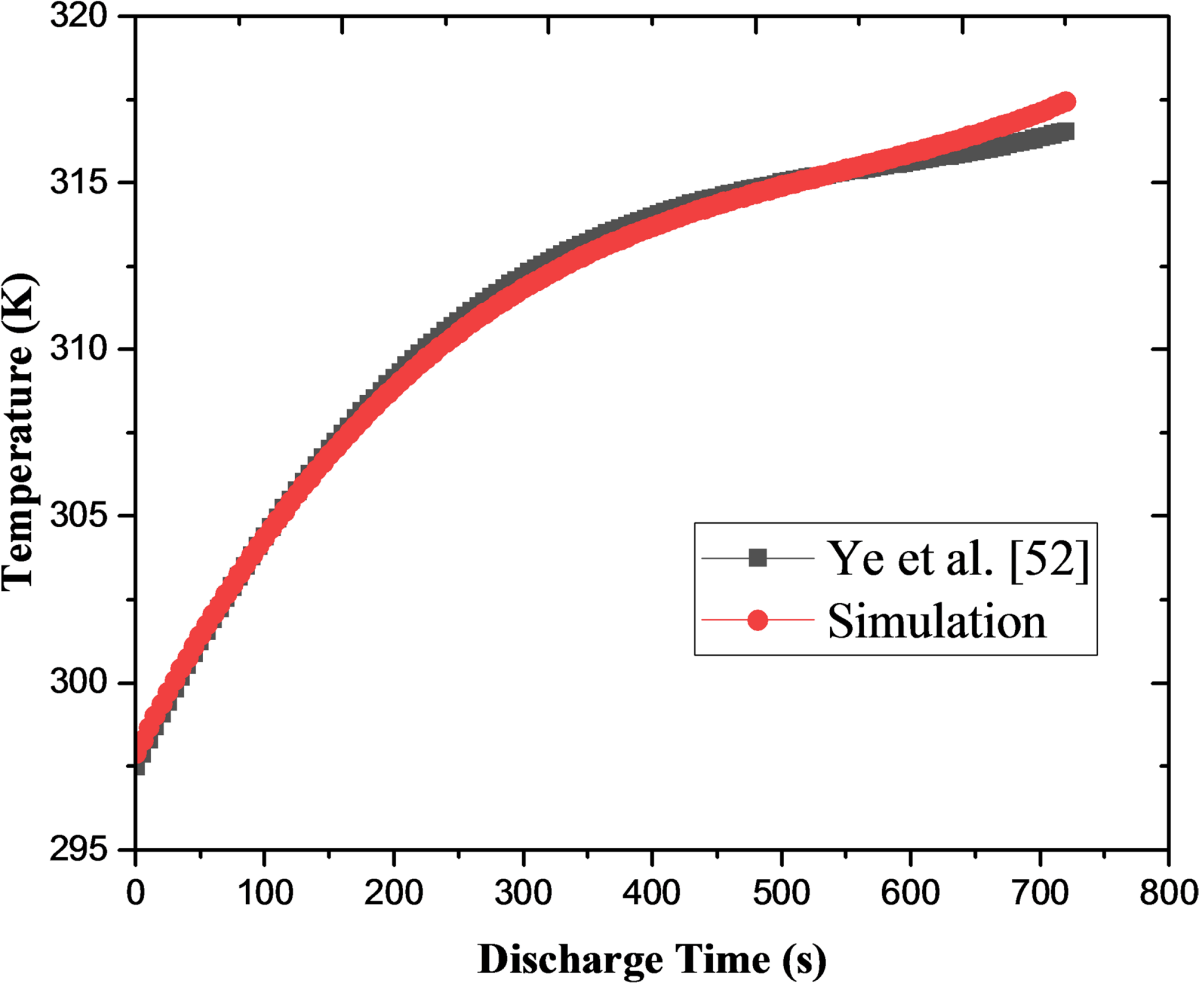
Validation of the temporal variation of maximum cell temperature with the experimental data^52^.

With the transient heat generation characteristics of the cell at 5 C, a user-defined function (UDF) is used to provide the heat generated per unit volume using the heat generation characteristics obtained from an experimental study^52^ With three sides of the cell insulated and one side having a convective heat transfer coefficient, as shown in Fig. 4, it is found that the transient variation of the maximum temperature of the cell follows the experimental characteristic curve. The temporal variation in the maximum temperature of the cell is then studied, and it is observed that the maximum deviation is less than 6% in the predicted cell temperature (Fig. 5).

**Fig. 6:**
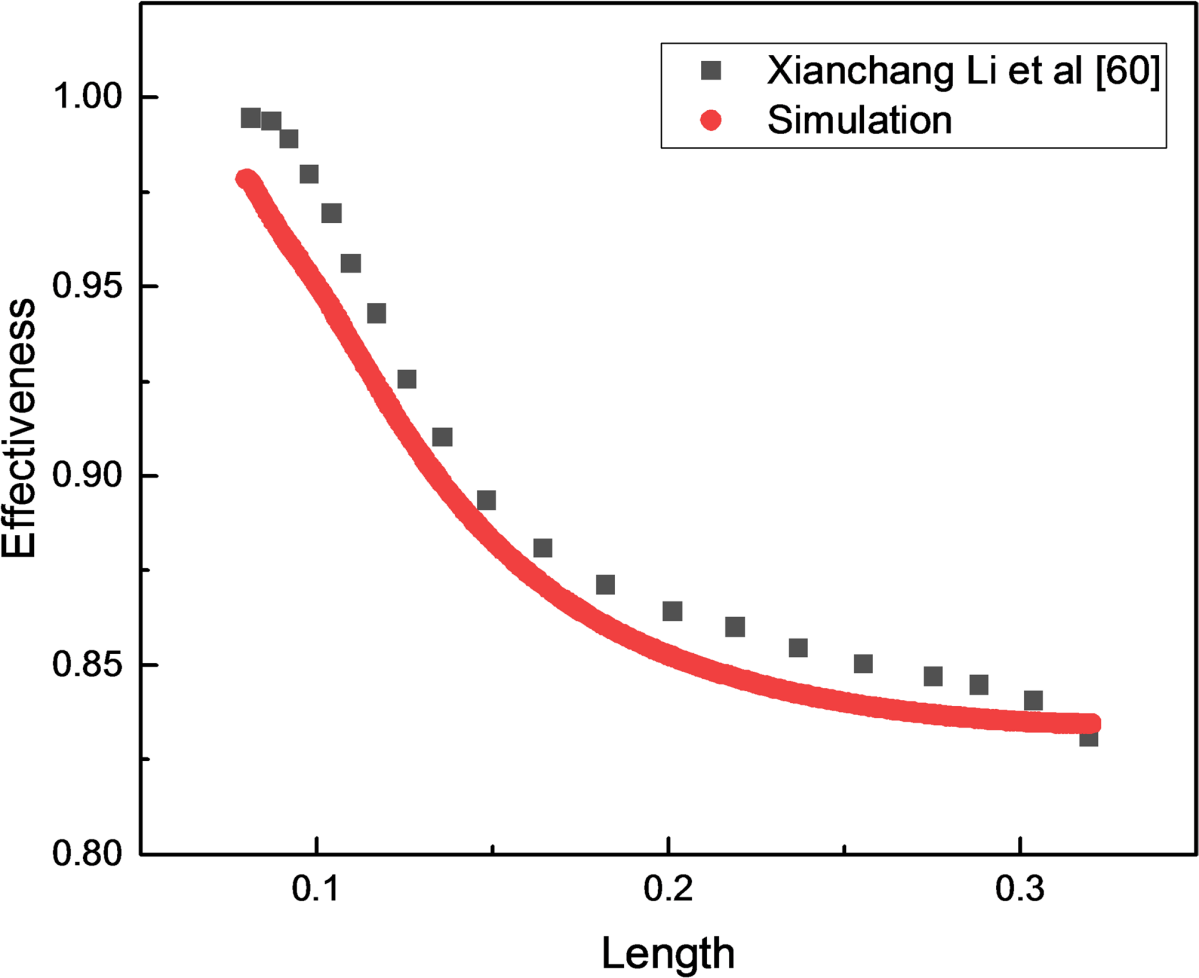
Validation of the effectiveness of mist cooling with experimental data.^60^

The mist cooling methodology is validated with experimental results for a duct cooling with a slot jet near the entrance of the mainstream (hot gases), where the adiabatic cooling effectiveness(η) is used to examine the performance of the mist coolin^60^. A maximum deviation of less than 6% indicates that the mist cooling numerical methodology is reasonably accurate (Fig. 6).

21 $$\:\eta\:=\frac{{T}_{g}-{T}_{aw}}{{T}_{g}-{T}_{c}}$$

Where, $\:{T}_{g}$ is the mainstream hot gas inlet temperature, $\:{T}_{c}$ is the temperature of the coolant and $\:{T}_{aw}$ is the adiabatic wall temperature.

## Results and discussion

A numerical analysis is conducted to investigate the mist cooling performance of a prismatic cell battery pack with an 8S1P configuration, operating at a discharge rate of 5 C and producing a nominal power of 1280 W at various locations and multiple injection points. To begin with, for a fixed air flow rate, mist loading fraction, water droplet diameter and flow rate, the study examines the performance of single mist injection at the bottom of the duct. Further performance with multiple injections is studied and compared to. The results are presented in the form of characteristic plots, including the transient variation of the maximum battery pack temperature and contour plots that display cell surface temperature distribution, coolant temperature, and water vapour distribution within the battery pack.

**Fig. 7:**
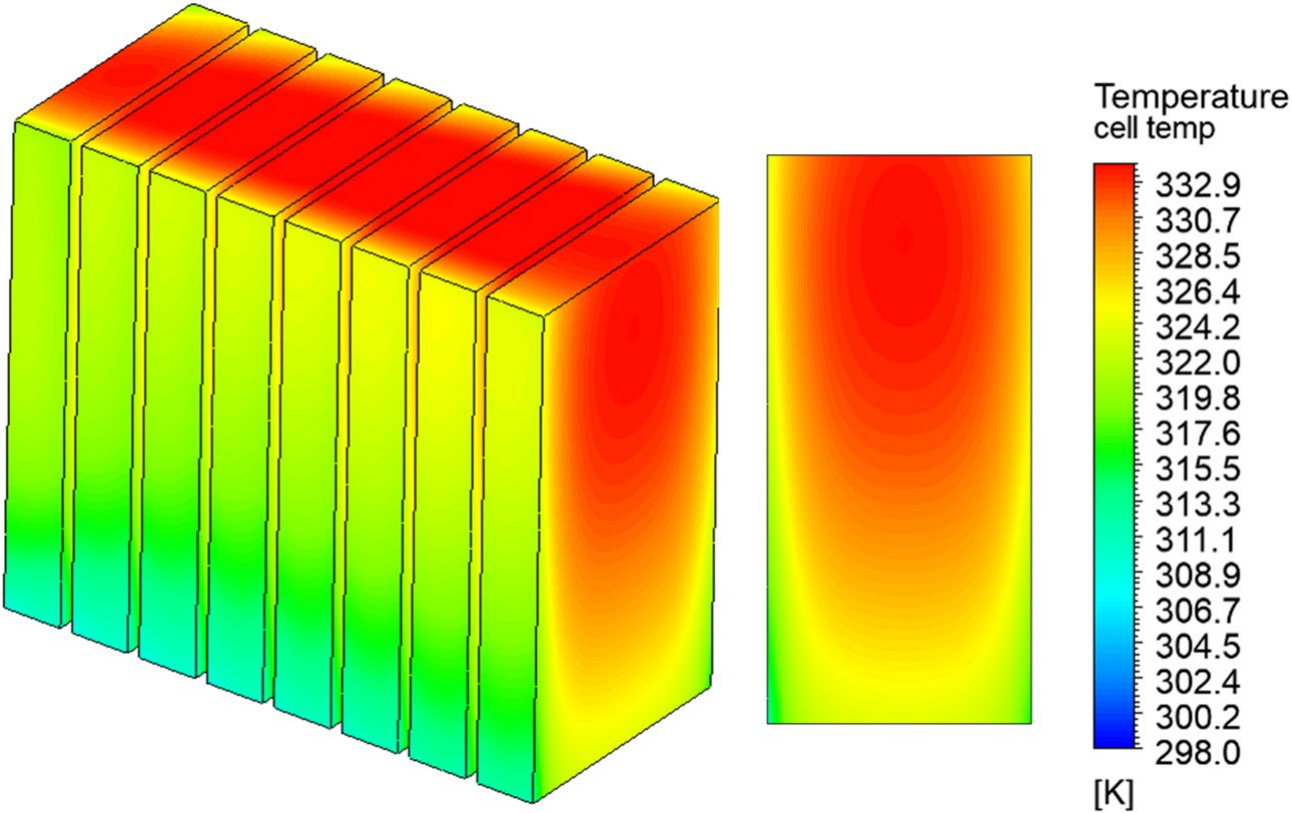
Spatial variation of temperature in the battery pack for air cooling.

**Fig. 8:**
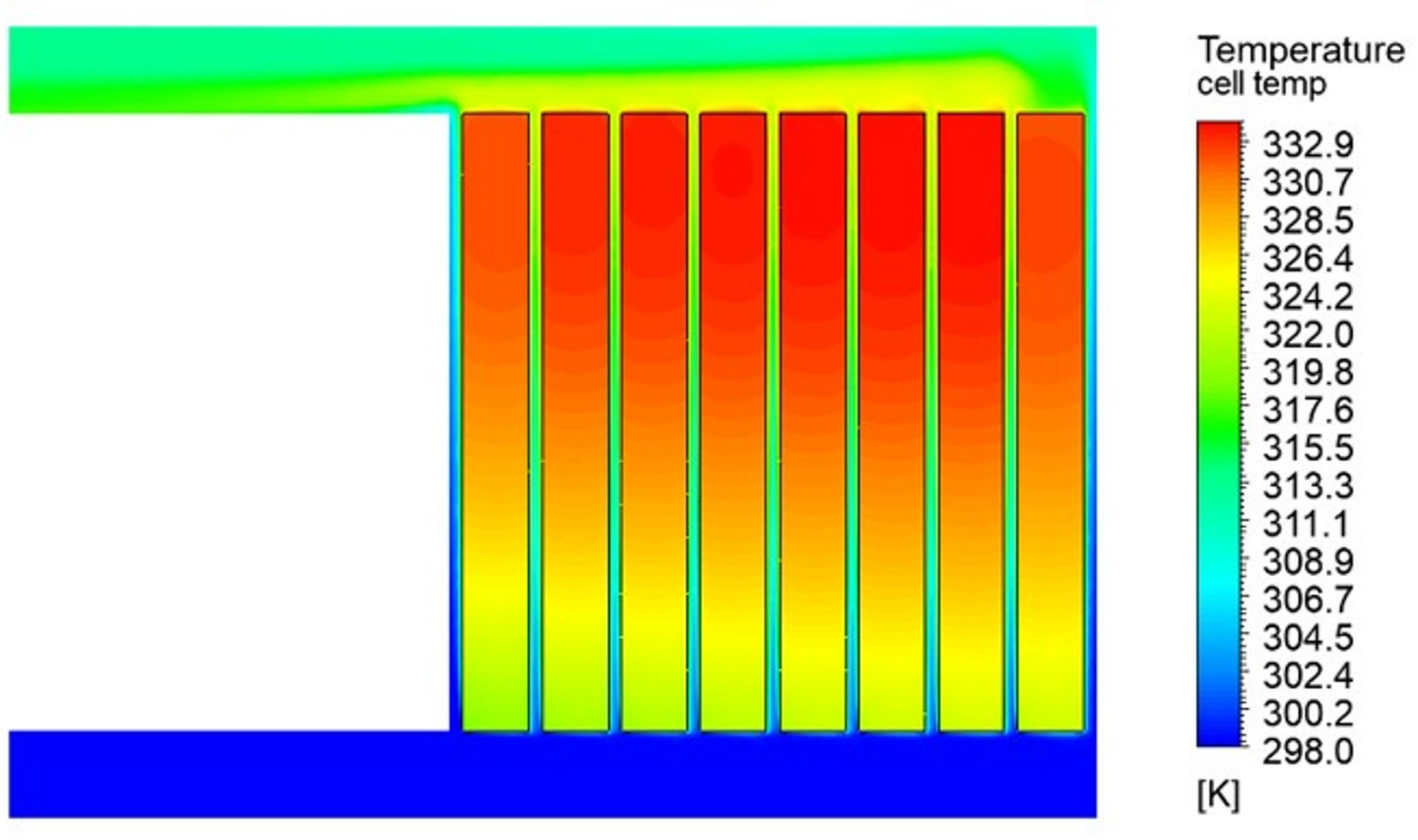
Spatial variation of temperature in the midplane of the battery pack for air cooling.

From the cell surface temperature distribution for the prismatic battery module under pure air cooling at a discharge rate of 5 C and an air mass flow rate of 0.0135 kg s⁻¹, it is observed that maximum cell surface temperature reaches approximately 62 °C, with the hottest regions located near to the outlet of the cooling domain (Fig. 7). The temperature difference between the hottest and coolest regions across the pack indicated substantial thermal non-uniformity owing to limited heat capacity of air and the development of thick thermal boundary layers over the large planar surfaces of prismatic cells towards the flow outlet. The corresponding mid-plane temperature contours in Fig. 8 further reveal a strong temperature gradient along the flow direction. Near the inlet, cells have temperatures of 44–46 °C, whereas towards the outlet, cells approach 60 °C, confirming cumulative heat accumulation as the cooling air progresses through the channel. Due to the lower thermal capacity and boundary-layer development, the air temperature in the intercell space increases in the flow direction, leading to a decrease in the convective heat transfer rate. This results in higher surface temperatures in the upper half of the cells.

**Fig. 9:**
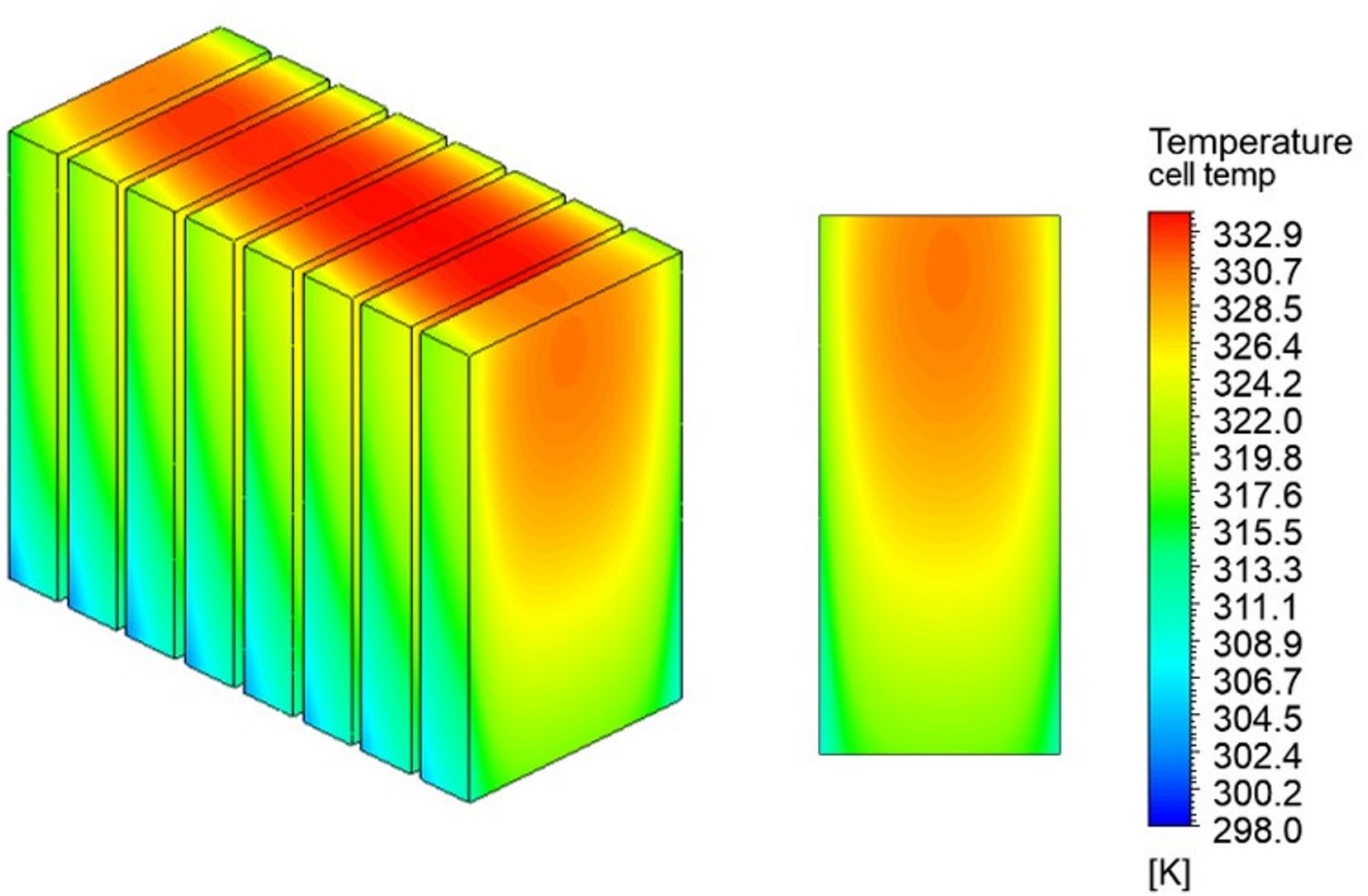
Spatial variation of temperature in the battery pack for mist cooling with single mist injection

When mist-assisted cooling is introduced through a single bottom injection, a clear modification in the thermal field is observed in the cell surface temperature contours (Fig. 9). The maximum cell surface temperature reduces to approximately 59 °C, representing a reduction of nearly 2 °C relative to the air-cooled case for the same mass flow rate. The high-temperature regions near the upper portion of the cells are significantly reduced, resulting in a lower temperature gradient. This behaviour indicates that droplets injected are effectively convected along the primary flow direction, allowing sustained evaporation as the air progresses through the cooling domain. The latent heat absorbed during droplet evaporation enhances the air’s sensible cooling capacity, thereby delaying boundary layer thickening and reducing cumulative heat accumulation near the domain outlet. The corresponding mid-plane temperature contours for bottom mist injection (Fig. 8) further support this observation.

**Fig. 10:**
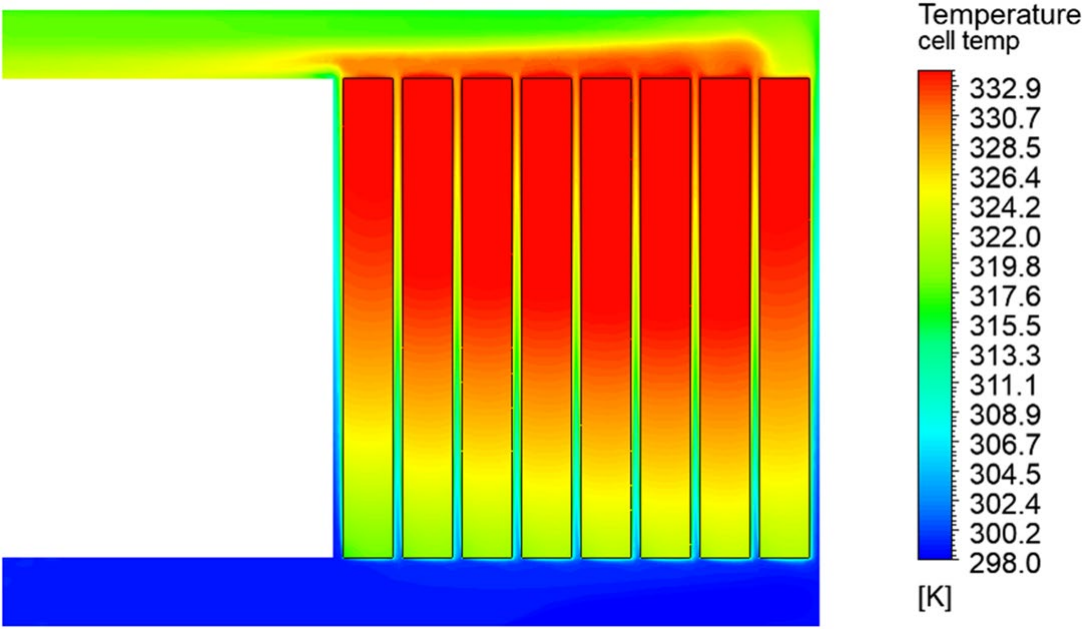
Spatial variation of temperature in the midplane of the battery pack for mist cooling with single mist injection.

Unlike the air-cooled case, where the mid-plane temperature rises monotonically along the flow direction, the mist-cooled configuration exhibits a moderated increase in temperature. Near the inlet, cell temperatures range from 40 to 42 °C, while those closer to the outlet are approximately 60 °C. Although single-point mist injection substantially improves the thermal performance compared to pure air cooling, the temperature contours indicate that cooling effectiveness remains spatially non-uniform, particularly toward the downstream regions. This limitation is overcome by employing multiple mist injection locations along the bottom of the domain. The corresponding mid-plane temperature contours in Fig. 10 further reveal a strong temperature gradient along the flow direction. Near the inlet, cells have a temperature in the range of 38 to 40 °C, whereas towards the outlet, cells approach 58 °C, confirming cumulative heat accumulation as the cooling air progresses through the channel.

**Fig. 11:**
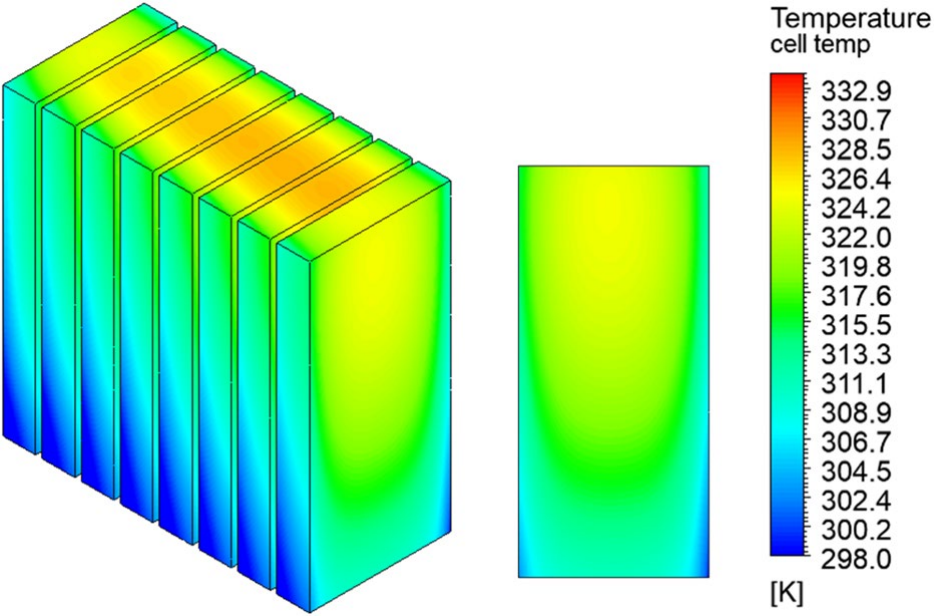
Spatial variation of temperature in the battery pack for mist cooling with multiple mist injection.

**Fig. 12:**
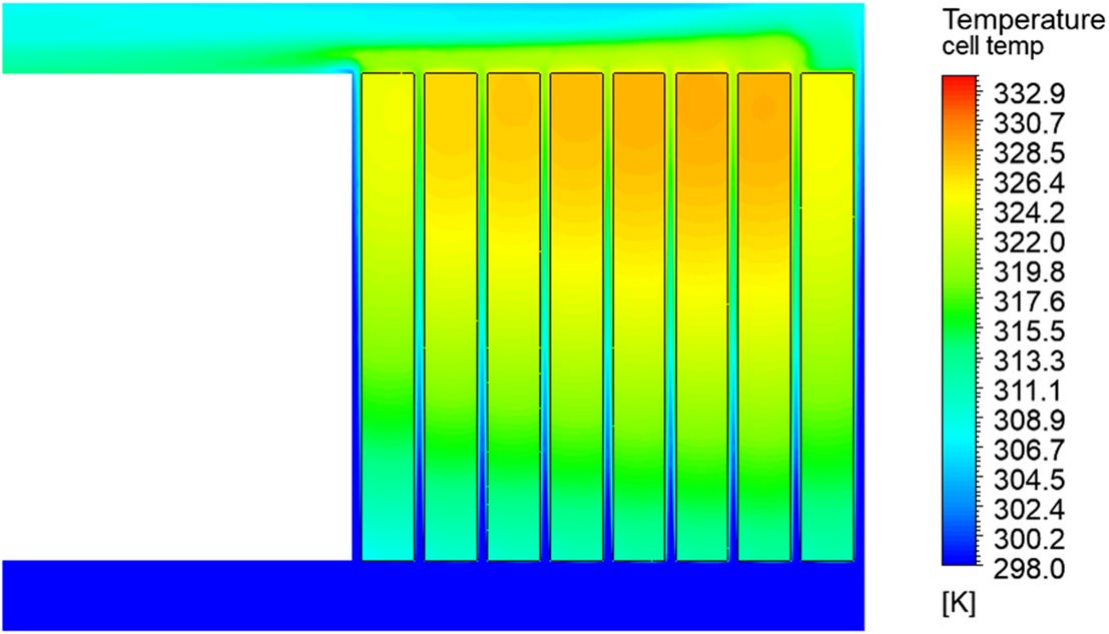
Spatial variation of temperature in the midplane of the battery pack for mist cooling with multiple mist injection.

**Fig. 13:**
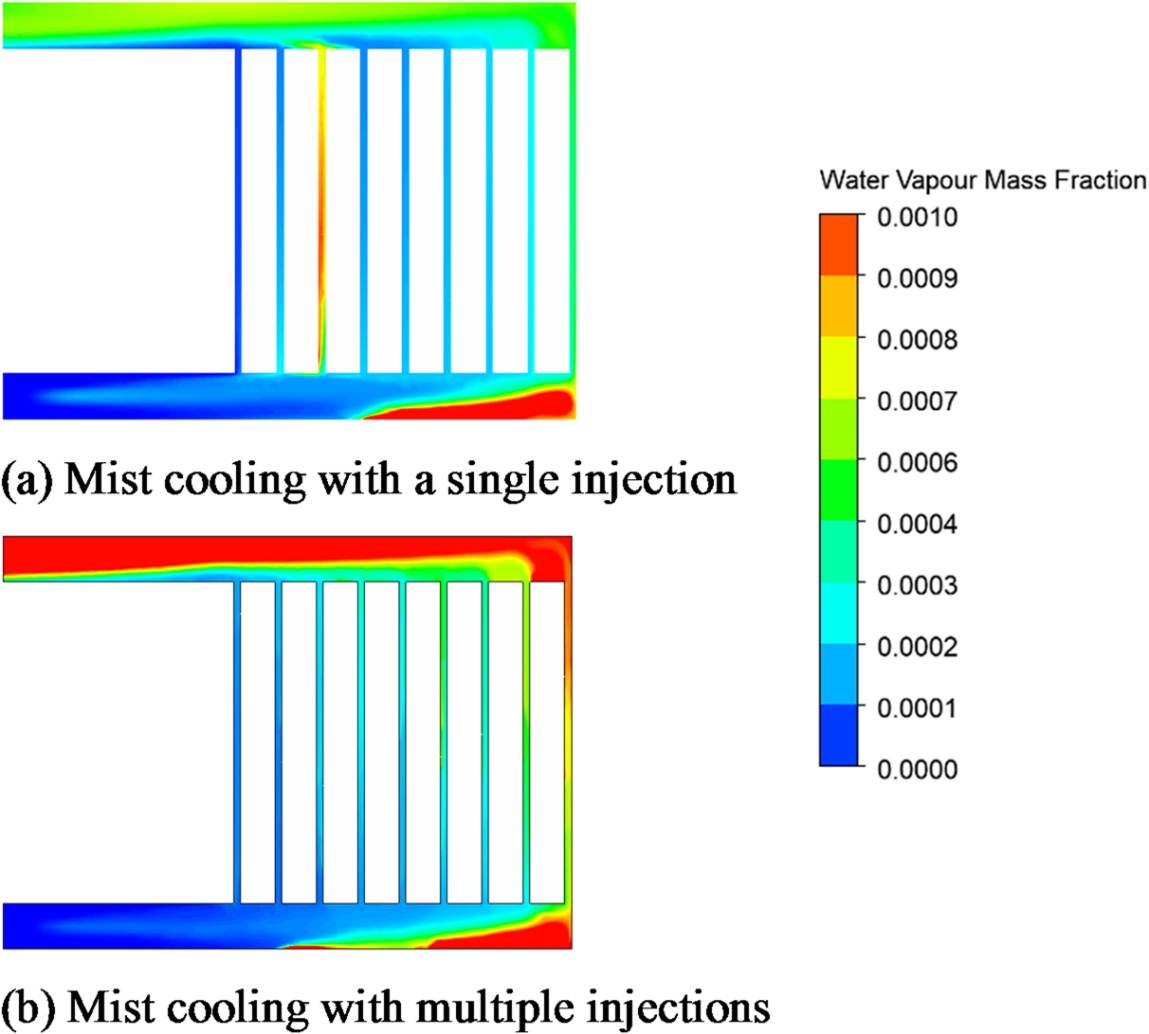
Water vapour mass fraction contours within the flow domain. (**a**) Mist cooling with a single injection, (**b**) Mist cooling with multiple injections.

**Fig. 14:**
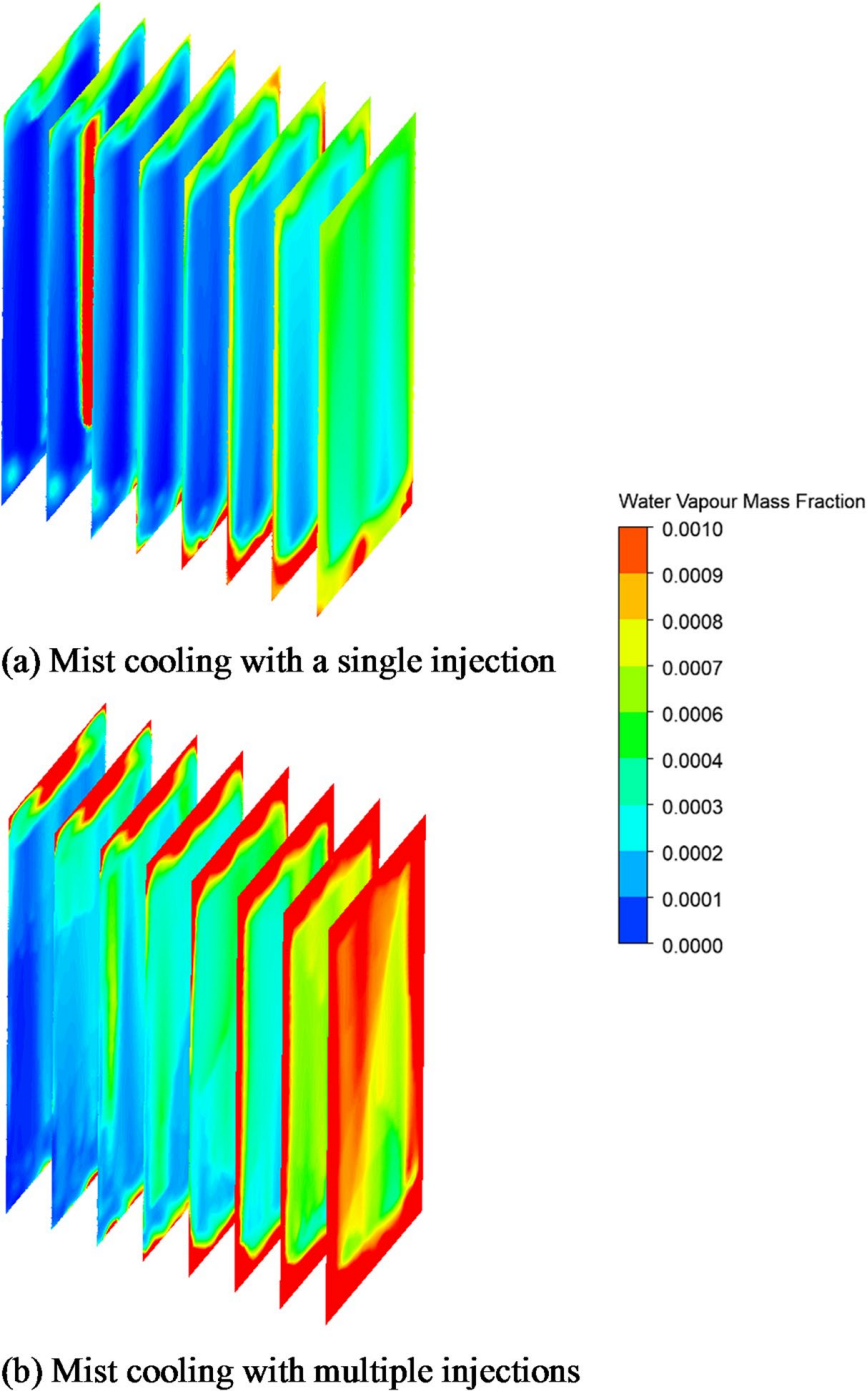
Water vapour mass fraction contours in the interspacing between cells.

In the multiple-injection configuration, the maximum cell surface temperature is further reduced to approximately 50–52 °C, corresponding to a reduction of nearly 10 °C compared to air cooling and 7 °C lower than the single-injection case (Fig. 11). More importantly, the temperature difference across the battery pack is now reduced, indicating a substantial improvement in thermal uniformity relative to conventional air cooling. The reduction in both peak temperature and temperature spread demonstrates that multiple injection ensures effective cooling not only near the injection region but also throughout the entire flow domain. The superior performance of multiple mist injection is further confirmed by the mid-plane temperature contours (Fig. 12). Unlike the single-injection case, the multiple-injection configuration exhibits an almost uniform mid-plane temperature distribution along the flow direction owing to pre-cooled air, even in the inter-cell spacing, resulting in reduced boundary layer thickness and higher convective heat transfer coefficient. The mechanisms governing the enhanced cooling performance of mist-assisted configurations can be clearly understood from the water vapour mass-fraction contours within the flow domain and the intercell spacing, presented in Figs. 13 and 14, respectively. These contours directly reveal the spatial extent and effectiveness of droplet evaporation, which supports the latent heat removal responsible for the observed temperature reductions.

For the single bottom mist injection case, the vapour mass-fraction contours within the flow domain (Fig. 13) show that evaporation is initiated near the injection location and remains dominant in the upstream and mid-sections of the channel. As the air–mist mixture progresses downstream, the vapour concentration increases gradually, indicating continuous but progressively weakening evaporation. This accumulation of vapour reduces the local vapour-pressure gradient between the droplets and the surrounding air, thereby diminishing the evaporation driving potential toward the outlet. As a result, although a single injection reduces the peak temperature by 2 to 3 °C relative to air cooling, it cannot fully suppress downstream heat accumulation, as shown in the temperature contours.

This limitation is further highlighted by the vapour mass-fraction distribution within the inter-cell spacing shown in Fig. 14. For single injection, higher vapour concentrations are primarily confined to regions closer to the injection side and near the lower portions of the cells, while comparatively lower vapour levels persist across other inter-cell gaps and along the upper cell height. This spatially non-uniform vapour distribution indicates that evaporation is localised rather than distributed, leading to uneven latent heat absorption and residual thermal non-uniformity across the battery pack. In contrast, the multiple mist injection configuration exhibits a fundamentally different vapour transport behaviour. As shown in Fig. 13, the vapour mass-fraction field becomes more evenly distributed along the entire flow direction due to the repeated introduction of fresh droplets at multiple axial locations. Instead of a monotonic vapour buildup, evaporation is sustained throughout the channel, preventing premature saturation of the carrier air and maintaining a favourable evaporation driving force even in downstream regions of the domain.

The superiority of this configuration is most evident in the inter-cell vapour contours (Fig. 14), where elevated vapour mass fractions are observed consistently across all inter-cell gaps and over the full domain height. This confirms that droplets effectively penetrate into the interspacing between adjacent prismatic cells, ensuring uniform latent heat absorption where convective cooling is otherwise weakest. Such spatially distributed evaporation directly explains the substantial improvement in thermal uniformity and the additional reduction in peak temperature achieved with multiple injection. From a physical perspective, these observations clarify that single injection configurations are inherently limited, despite their effectiveness relative to air cooling. In a single injection, evaporation is front-loaded, and the cooling potential diminishes downstream due to vapour accumulation and boundary-layer redevelopment. Multiple injection overcomes this constraint by continuously replenishing droplets, periodically disrupting thermal boundary layers, and sustaining evaporation over the entire battery pack.

**Fig. 15:**
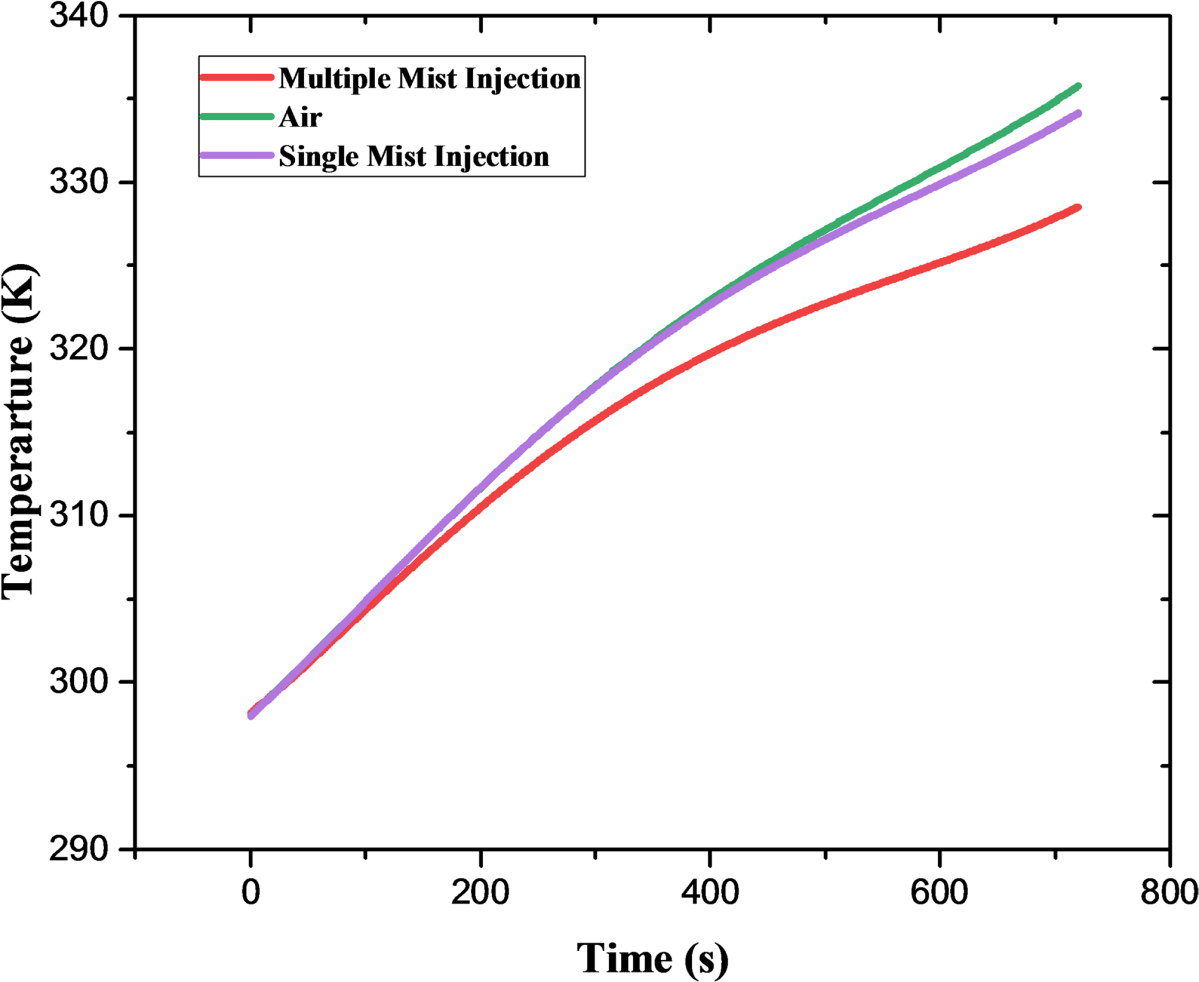
Variation of the maximum temperature of the battery with depth of discharge for a 5 C discharge rate.

The transient thermal response of the battery pack is further illustrated by studying the maximum temperature variation with depth of discharge (Fig. 15) under sustained 5 C discharge. For the air-cooled case, the temperature–time plot shows a rapid initial rise followed by a gradual, continuous increase, with no clear plateau reached during the discharge. This behaviour reflects the limited heat removal capacity of air, where the rate of heat generation exceeds the combined sensible heat removal and convective transport, leading to cumulative thermal energy storage within the cells. In contrast, both single-point and multiple-mist injection cases exhibit noticeably different temporal behaviour. With single mist injection, the temperature rise rate is significantly reduced after the initial transient, and the temperature curve approaches a quasi-steady state, with a lower rate of temperature rise near the end of the discharge. This indicates that latent heat absorption through droplet evaporation begins to effectively balance the heat generation rate. However, evaporation effectiveness gradually diminishes as vapour accumulates downstream, resulting in only a 2–3 °C drop, consistent with the vapour-contour observations discussed earlier.

The multiple mist injection configuration exhibits the most stable thermal response among the cooling cases. After an initial rise, the cell temperature reaches a near-steady value, and the absence of a sustained temperature drift indicates that distributed evaporation continuously offsets heat generation, preventing long-term thermal accumulation. This transient behaviour highlights that multiple injection is not only superior in reducing peak temperature but also more effective in maintaining long-term thermal stability under high-rate operation. Even though the maximum temperature drops, the temperature non-uniformity remains higher after a single mist injection. Under this scenario, the multiple mist injection case significantly reduces the temperature non-uniformity.

Taken together, the maximum temperature and temperature uniformity plots provide complementary validation of the cooling mechanisms discussed earlier. While peak temperature reduction indicates the effectiveness of a cooling strategy, the reduction in temperature spread directly reflects improvements in thermal uniformity, which is a critical parameter for mitigating uneven ageing and capacity fade in prismatic battery packs. The results clearly demonstrate that mist-assisted cooling, particularly with multiple injections, delivers both rapid thermal stabilisation and superior cell-to-cell temperature uniformity.

Finally, at the system level, the improved thermal performance achieved with mist-assisted cooling is obtained without a significant increase in pumping power. The primary airflow rate remains unchanged, and the additional energy requirement is limited to the operation of the mist injectors, which is typically negligible compared to the power required for high-speed air blowers or liquid coolant pumps. Previous studies on mist and spray-assisted cooling have shown that the auxiliary power consumption of injectors constitutes only a small fraction of the total cooling power budget, while delivering disproportionately large thermal benefits^45^. Consequently, mist-assisted air cooling represents a thermally effective and energetically efficient enhancement over conventional air cooling, making it a promising candidate for high-rate prismatic battery thermal management.

## Conclusion

This study numerically investigated mist-assisted air cooling as an enhancement to conventional forced air cooling for a high-discharge (5 C) prismatic LiFePO₄ battery pack. A transient three-dimensional CFD framework incorporating detailed spray dynamics, droplet breakup, evaporation, and two-way phase coupling was employed to systematically compare pure-air cooling with single- and multiple-mist injection strategies. The analysis focused on peak temperature reduction, spatial temperature uniformity, vapour transport characteristics, and transient thermal stability under sustained high-rate operation. The key conclusions are summarised as follows:

- With air cooling, the prismatic battery pack exhibited a high maximum temperature of approximately 62 °C with severe thermal non-uniformity, arising from limited air heat capacity and progressive boundary-layer development along the flow direction.
- The introduction of single-point mist injection reduced the peak cell temperature by 2–3 °C compared to air cooling, confirming the beneficial role of latent heat absorption through droplet evaporation. However, vapour accumulation downstream weakened evaporation potential, resulting in persistent spatial non-uniformity.
- The multiple mist injection configuration delivered the best overall thermal performance, reducing the maximum cell temperature by nearly 10 °C compared to air cooling and 6 °C relative to single injection.
- Vapour mass-fraction contours demonstrated that distributed injection sustains evaporation throughout the entire flow domain and maintains high convective effectiveness even in downstream regions, reducing temperature non-uniformity compared to air cooling.
- The additional energy demand is limited to mist injectors, which are negligible compared to the power required for the blower or liquid pumping. Consequently, mist-assisted air cooling offers a lightweight, compact, and energy-efficient alternative to liquid-based BTMS, while overcoming the fundamental heat capacity limitations of air cooling for high-rate prismatic cells.

Future work should extend the present framework to incorporate climate-dependent ambient conditions and experimental validation. Further optimisation of droplet size distribution, injection spacing, and adaptive control strategies based on real-time thermal feedback would enable practical deployment of mist-assisted cooling in next-generation electric vehicle battery packs. Extending the approach to larger battery packs and fast-charging scenarios would further support EV thermal safety. The findings directly contribute to Sustainable Development Goal (SDG) 7: Affordable and Clean Energy, by improving battery efficiency, safety, and reliability, key enablers for sustainable transportation systems.

## Data Availability

The data that support the findings can be provided upon request to the corresponding author.

## List of symbols

T: Temperature (K)
k: Thermal conductivity (W·m⁻¹·K⁻¹)
ρ: Density (kg·m⁻³)
c_p_: Specific heat capacity (J·kg⁻¹·K⁻¹)
q̇: Volumetric heat-generation rate (W·m⁻³)
I: Current (A)
V: Cell voltage (V)
Q_nom_: Nominal cell capacity (Ah)
Q_ref_: Reference capacity (Ah)
ṁ_p_: Particle mass flow rate (kg·s⁻¹)
d_p_: Droplet diameter (m)
A_p_: Droplet surface area (m²)
u_p_: Particle velocity (m·s⁻¹)
u: Fluid velocity (m·s⁻¹)
F_p_: Net force acting on particle (N)
m_p_: Droplet mass (kg)
h: Convective heat-transfer coefficient (W·m⁻²·K⁻¹)
D: Mass diffusion coefficient (m²·s⁻¹)
ΔT: Temperature difference (K)

## Greek symbols

µ: Dynamic viscosity (Pa·s)
τ: Shear/deformation tensor
η: Adiabatic cooling effectiveness
λ: Thermal diffusivity (m²·s⁻¹)
β: Evaporation mass-transfer coefficient
ω_v_: Water vapour mass fraction
φ: Relative humidity (%)
K_n_: Knudsen number

## Dimensionless numbers

Re: Reynolds number
Nu: Nusselt number
Sh: Sherwood number
C_d_: Drag coefficient

## Subscripts

p: Particle/droplet
f: Fluid/air
in: Inlet
out: Outlet
max: Maximum value
vap: Vapour phase
wall: Cell or channel wall
ref: Reference condition

## Acronyms

BTMS: Battery Thermal Management System
CFD: Computational Fluid Dynamics
NTGK: Newman–Tiedemann–Gu–Kim model
DPM: Discrete Phase Model
TAB: Taylor Analogy Breakup model
PCM: Phase Change Material
EV: Electric Vehicle
SOC: State of Charge
SOH: State of Health
RH: Relative Humidity
